# Sleep-related Eating Disorder in a Patient with Parkinson's Disease

**DOI:** 10.7759/cureus.3345

**Published:** 2018-09-22

**Authors:** Harleen Kaur, Muhammad Umair Jahngir, Junaid H Siddiqui

**Affiliations:** 1 Neurology, Univeristy of Missouri, Columbia, USA; 2 Neurology, University of Missouri, Columbia, USA

**Keywords:** parkinson\'s disease, parasomnias, sleep related eating disorder, sred, parkinson's disease

## Abstract

Sleep disorders constitute a major aspect of the non-motor symptoms of Parkinson’s disease (PD). Rapid eye movement (REM) behavior disorders are the most frequently experienced parasomnias in patients with PD. Non-REM sleep disorders like confusional arousals, sleep terrors, sleepwalking, and sleep-related eating disorder (SRED) are also associated with PD. Parasomnias can affect the quality of life of the patients as well as the night time sleep of their bed partners. Hence, it is important for physicians to recognize the occurrence of parasomnias in PD. We report an unusual case of PD with SRED along with obstructive sleep apnea (OSA) and REM behavior disorder. To our knowledge, only two cases have been reported in the literature highlighting the association of SRED with PD. We also explain the different night-time eating disorders like nocturnal eating syndrome and binge eating syndrome, which can be seen in PD, and differentiate them from SRED.

## Introduction

Parkinson’s disease (PD) is a neurodegenerative disease with a spectrum of motor and non-motor symptoms. The non-motor symptoms of PD manifest as dysautonomia, cognitive changes, mood disorders, and sleep-related disorder. Parasomnias are frequently experienced in patients with PD as non-motor symptoms. Rapid eye movement (REM) sleep behavior disorders (RBD) is considered the most common parasomnia. The non-REM sleep disorders, including sleepwalking, sleep terrors, confusional arousals, and sleep-related eating disorder, are also less frequently noted. The PD-related parasomnias have a high prevalence of 98% [1-2]. They not only affect the quality of life of patients but also disrupt the night-time sleep of their bed partners and may even result in injury. There is ample literature indicating the early occurrence of RBD in PD patients, but less is known about the association of non-REM sleep disorders, mainly sleep-related eating disorder (SRED) with PD. To our knowledge, until now, only two cases have reported the association of SRED in PD patients. We report an unusual case of PD with obstructive sleep apnea (OSA) and the typical symptoms of SRED. The patient was clinically evaluated by a movement disorder specialist and a sleep specialist to address the underlying concerns.

## Case presentation

Our patient is a 56-year-old, left-handed female, with a two-year history of PD. Her initial symptoms included tremors in the left hand, along with complaints of micrographia, hypophonia, and fatigue. Her initial management included an incremental dose of Sinemet (25/100 mg) to which she developed severe nausea, necessitating its discontinuation. Subsequently, she was prescribed an incremental dose of pramipexole 0.125 mg twice a day by a neurologist at a community hospital. She was later advised to reduce the dose of pramipexole to half a tablet twice a day because of the side-effects of nausea, dizziness, sedation, and increased urinary frequency. She further complained of persisting symptoms of polyuria, frequent leg cramps, and lack of a feeling of well-being on pramipexole. In lieu of the persisting symptoms, pramipexole was discontinued. During this course, amantadine was also tried for tremors but discontinued because of worsening tremors. A trial of propranolol was also ineffective. She also used cannabinoid oil and medical massage but did not help her symptoms. Her diagnostic workup also included magnetic resonance imaging (MRI) of the brain and whole spine. Cerebrospinal fluid (CSF) was also obtained and reported as normal in the past.

She was diagnosed with OSA in the past but was unable to tolerate continuous positive airway pressure machine (CPAP). She also had a history of REM sleep behavior disorder along with episodes of somnambulism (sleepwalking) and bruxism. In her later clinic visits, she reported a new onset of a sleep-related eating disorder as described by her husband. She had an episode of unconsciously walking in the kitchen, eating her husband’s chocolate, and going back to bed. She reported another similar episode of eating her husband’s cereal unconsciously at night, which she had apparently disliked. She denied any episodes of binge eating during the day or night time. There was no history of episodes of consciously waking up at night to consume food. There was no history of hallucinations or cognitive dysfunction.

Her past medical and surgical history includes secondary hypothyroidism for which she is taking levothyroxine, L4-L5 laminectomy, thyroidectomy, and hysterectomy. She is on nortriptyline for anxiety and depression.

Further, she noticed a worsening of her PD symptoms. Her unified Parkinson’s disease rating scale (UPDRS) deteriorated from 30 to 41 over nine months. Her tremor worsened on the left side and gradually progressed, involving the right side.

## Discussion

Sleep disorders are common non-motor symptoms of Parkinson’s Disease with a high prevalence of up to 98% [1-2]. PD is a gradually progressive neurodegenerative disease affecting the ‘ascending’ control of sleep state transition and ‘descending’ control of movement and muscle tone thereby resulting in parasomnias and sleep-related disorders [3]. Sleep disorders in PD patients manifest largely as REM sleep behavior disorder (RBD), in which the patients act out their dreams and appear to kick, punch, or scream. The prevalence of RBD in PD patients ranges from 22.5%-85%[4-5]. Non-rapid eye movement (NREM) sleep-related parasomnia also occur with PD. They can manifest as confusional arousals, sleep terrors, sleep-related hallucinations, sleepwalking, or sleep-related eating disorders. Ylikoski et al. performed a questionnaire study in patients with PD and found 39% of patients with RBD had coexisting symptoms of other parasomnias, including nightmares (17.2%), hallucinations (15.3%), night terror (3.9%), and enuresis (21%) [5]. The coexistence of RBD and NREM behavior disorders is termed overlapping syndrome and can be seen in patients with PD. These nighttime problems can affect the quality of life of the PD patients, resulting in fatigue, impaired daytime functioning, and disturbing the night-time sleep of the caregivers or bed-partners.

There is literature describing the association of PD with RBD but not much is available regarding the association of PD with SRED. We report a patient with PD who was unable to tolerate dopaminergic medications along with a history of RBD, SRED, and OSA. To our knowledge, there are two case reports so far reporting PD with SRED. One of the case reports highlighted the casual association between the dose of pramipexole and SRED and improvement in symptoms on reducing the dose. The other case highlighted the association of an overlapping syndrome with OSA and an improvement in symptoms of SRED was noted on managing the OSA with CPAP [6].

SRED may be associated with sleepwalking, periodic limb movements, OSA, or restless leg syndrome (RLS) [7-8] or maybe a possible side-effect from the intake of medications like antidepressants, olanzapine [9], risperidone [10], lithium, triazolam, and zolpidem [11]. It is important to differentiate SRED from nocturnal eating syndrome (NES) and compulsive binge eating as a result of impulse control disorders, which can also be seen in PD. While SRED involves recurrent episodes of eating or drinking peculiar food combination or even the ingestion of toxic substances in a relatively unconscious state during the sleep period, NES is considered a delayed circadian consumption of food where the patient wakes up at night and consciously binges on food, resulting in next morning anorexia and sleep fragmentation. NES is associated with conscious evening hyperphagia and sleep onset insomnia [12-13]. The association between SRED and NES is unclear.

Compulsive binge eating is an impulsive behavior resulting in the conscious consumption of large amounts of normal food. Studies have shown an association of compulsive binge eating in patients with PD on dopamine agonists like pramipexole [14]. Also, there is a reported association of SRED with a dose of pramipexole. Provini et al. observed in their study that lowering the dose of pramipexole (0.36 mg/day) reduced the symptoms of SRED[15]. However, this placebo control trial did not include PD patients. Another case-control study suggested that patients who received dopaminergic therapy for RLS had a higher risk of developing SRED[14].

In our case study, the patient was unable to tolerate dopamine agonist and SRED symptoms emerged long after the discontinuation of pramipexole. Even though our patient has OSA, she refused to use a CPAP machine at night and hence the improvement in symptoms of SRED could not be determined, as in other case reports.

## Conclusions

Sleep-related disorders are a common manifestation of the non-motor symptoms of PD. These symptoms can disrupt the quality of life of the patient and their bed partners and may even result in injury. Hence, it is vital for medical personnel in clinical practice to be able to identify parasomnias in PD and differentiate between various subtypes. SRED is an unusual non-REM parasomnia that can be seen in PD. The early detection of parasomnia can improve the well-being and safety of the patients as well as improve the quality of sleep of their bed partners.
